# Cross-species transferability of EST-SSR markers developed from the transcriptome of *Melilotus* and their application to population genetics research

**DOI:** 10.1038/s41598-017-18049-8

**Published:** 2017-12-20

**Authors:** Zhuanzhuan Yan, Fan Wu, Kai Luo, Yufeng Zhao, Qi Yan, Yufei Zhang, Yanrong Wang, Jiyu Zhang

**Affiliations:** State Key Laboratory of Grassland Agro-ecosystems, Key Laboratory of Grassland Livestock Industry Innovation, Ministry of Agriculture, College of Pastoral Agriculture Science and Technology, Lanzhou University, Lanzhou, 730020 China

## Abstract

*Melilotus* is one of the most important legume forages, but the lack of molecular markers has limited the development and utilization of *Melilotus* germplasm resources. In the present study, 151 M clean reads were generated from various genotypes of *Melilotus albus* using Illumina sequencing. A total of 19,263 potential EST-SSRs were identified from 104,358 unigene sequences. Moreover, 18,182 primer pairs were successfully designed, and 550 primer pairs were selected using criteria of base repeat type, fragment length and annealing temperature. In addition, 550 primer pairs were screened by using PCR amplification products and used to assess polymorphisms in 15 *M. albus* accessions. A total of 114 primer pairs were detected as being highly polymorphic, and the average polymorphism information content (*PIC*) value was 0.79. Furthermore, those 114 polymorphic primer pairs were used to evaluate the transferability to 18 species of the genus *Melilotus*, and 70 EST-SSR markers were found to be transferable among the 18 *Melilotus* species. According to the UPGMA dendrogram and STRUCTURE analysis, the 18 *Melilotus* species were classified into three clusters. This study offers a valuable resource for the genetic diversity and molecular assisted breeding of germplasm resources in the genus *Melilotus*.

## Introduction

*Melilotus* is one of the most important legume forages, and this genus is comprised of 19 annual or biennial species^1^. All species are native to Eurasia or North Africa^2^ and diploid with 16 chromosomes (2n = 16) and accessions of *Melilotus* have two pollination methods, self-pollination and cross-pollination^3^. Compared with most other pasture crops, members of the genus *Melilotus* have high seed yields and are adapted to harsh environmental conditions, such as drought, cold and high salinity^4–6^. In addition, it is important for agriculture and animal husbandry, as it is a green manure crop that can be used as a crop fertilizer^7^. As forage legumes, they have the ability to perform symbiotic nitrogen fixation with species of bacteria species^8^, and their nitrogen fixation rate is higher than those of many other legumes, making them beneficial for crop rotation^9^. Previous studies in *Melilotus* were mainly focused on cultivation techniques^10^, chemical composition^11^ and assessment of the agronomic and quality traits^12^. The genus *Melilotus* has a single inheritance system, and its relationships with alfalfa and clover were confirmed based on the phylogenetic tree^13^. However, the molecular markers for the genus *Melilotus* are limited, hindering its use in genetic and breeding studies. To date, only very limited information has been provided, form 9 SSRs to study the origins of the sweet clover invasion in Alaska^14^, and 18 SSRs to study the genetic diversity of different species of *Melilotus* ^7^. Developing some highly polymorphic EST-SSR markers would allow a better understanding of the genetic diversity in *Melilotus*, which could facilitate *Melilotus* breeding programs.

Simple sequence repeats (SSRs) are tandem repeated sequences comprising mono-, di-, tri-, tetra-, penta- or hexa-nucleotide units. Compared with other molecular markers, SSRs have high polymorphism, co-dominance, and locus specificity and are easy to detect^15^. SSRs are useful tools for studying genetic variation, genetic mapping, and molecular breeding^16–20^, and they have a high level of transferability between closely related species^21^. EST-SSRs are a type of molecular marker based on expressed sequence tags; compared with genomic-SSRs, EST-SSRs have a higher level of transferability across related species because EST-SSRs originate from the transcribed regions in genomes and possess conserved sequences among homologous genes^22^. At present, EST-SSR is widely used in plant genomics research, such as genetic map construction, comparative mapping, genetic diversity evaluation, germplasm identification, and phylogenetic and evolutionary studies^23–25^.

In this study, we aim to (1) develop EST-SSR markers for *M. albus* using the Illumina HiSeq. 2000 sequencing platform, (2) screen the 550 primer pairs that were selected based on the conditions of base repeat type, fragment length and annealing temperature, by using PCR and PAGE electrophoresis from fifteen accessions of *M. albus*, (3) detect the transferable and polymorphic EST-SSR markers for the genus *Melilotus*, and (4) reveal the population structure of *Melilotus* species.

## Results

### Sequencing and distribution of EST-SSR

In the present study, we generated 32,939,751, 31,176,000, 32,518,646, 31,470,600 and 35,446,843 raw reads using Illumina sequencing, which included the empty and low-quality reads from the *M. albus* genotypes N46, N47, N48, N49 and RPh, respectively. After rigorous quality control and data filtering, we generated 30,532,020, 28,785,103, 30,067,146, 29,041,739 and 32,634,517 clean reads, which were deposited into the NCBI SRA database, and obtained 154,458 transcripts and 104,358 unigenes. A total of 19,263 EST-SSR loci were detected from 104,358 unigene sequences, and 18,182 primer pairs were successfully designed. Of these unigenes, 3,063 unigenes contained more than one EST-SSR (Table S1). An average of one EST-SSR was found every 3.99 kb, and the frequency of SSRs was 14.60%. Among the 19,263 potential EST-SSRs, six types of motifs were identified: mononucleotides (12,052, 62.57%), dinucleotides (3,200, 16.61%), trinucleotides (3,654, 18.97%), tetranucleotides (319, 1.66%), pentanucleotides (29, 0.15%), and hexanucleotides (9, 0.05%). EST-SSRs with ten tandem repeats (27.58%) were the most common, and these were followed by eleven, five, six and seven tandem repeats, representing 13.42, 13.29, 11.74, and 5.48%, respectively, while the remaining tandem repeats each accounted for less than 5% of the EST-SSRs (Table 1).

**Table 1:** Distribution of EST-SSRs with different repeat types.

Repeats	Mononucleotide	Dinucleotide	Trinucleotide	Tetranucleotide	Pentanucleotide	Hexanucleotide	Total	Percentage (%)
5	0	0	2247	282	27	5	2561	13.29
6	0	1221	1002	34	2	3	2262	11.74
7	0	674	378	2	0	1	1055	5.48
8	0	540	24	0	0	0	564	2.93
9	0	409	0	0	0	0	409	2.12
10	5040	271	1	1	0	0	5313	27.58
11	2503	82	1	0	0	0	2586	13.42
12	1397	3	0	0	0	0	1400	7.27
13	952	0	0	0	0	0	952	4.94
14	685	0	0	0	0	0	685	3.56
15	456	0	0	0	0	0	456	2.37
16	340	0	0	0	0	0	340	1.77
17	238	0	0	0	0	0	238	1.24
18	181	0	0	0	0	0	181	0.94
19	108	0	0	0	0	0	108	0.56
20	88	0	0	0	0	0	88	0.46
21	43	0	0	0	0	0	43	0.22
22	16	0	0	0	0	0	16	0.08
23	4	0	1	0	0	0	5	0.03
24	1	0	0	0	0	0	1	0.01
Total	12052	3200	3654	319	29	9	19263	
Percentage (%)	62.57	16.61	18.97	1.66	0.15	0.05		

### Development, screening, and polymorphism of EST-SSR

**Figure 1:**
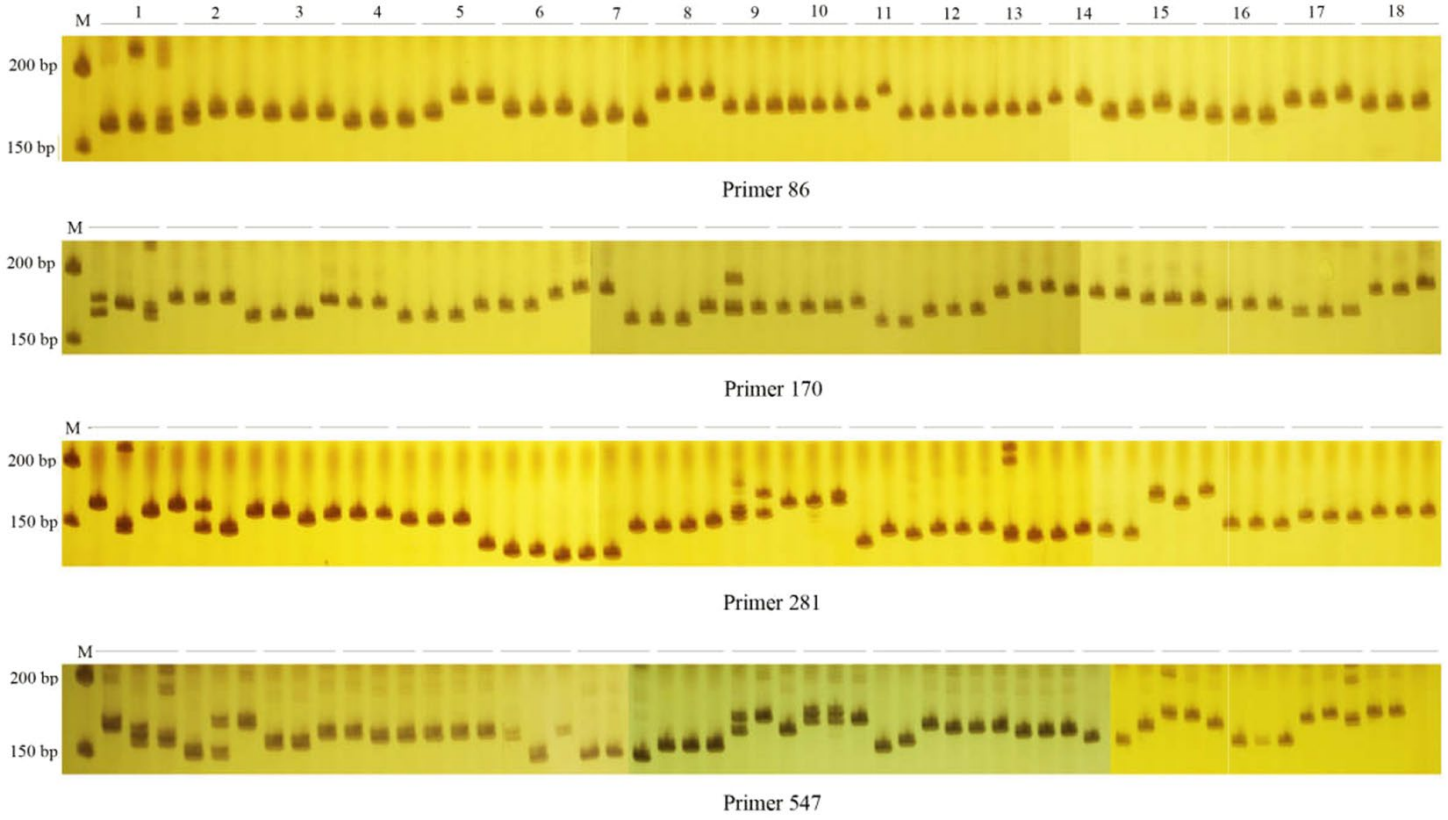
EST-SSR marker variations of 18 *Melilotus* species using primers 86, 170, 281 and 547. Each accession includes three individual plants; the letter ‘M’ denotes the molecular markers, which are 200 bp and 150 bp (top to bottom) with primer 86, primer 170, primer 281 and primer 547 (top to bottom).

**Figure 2:**
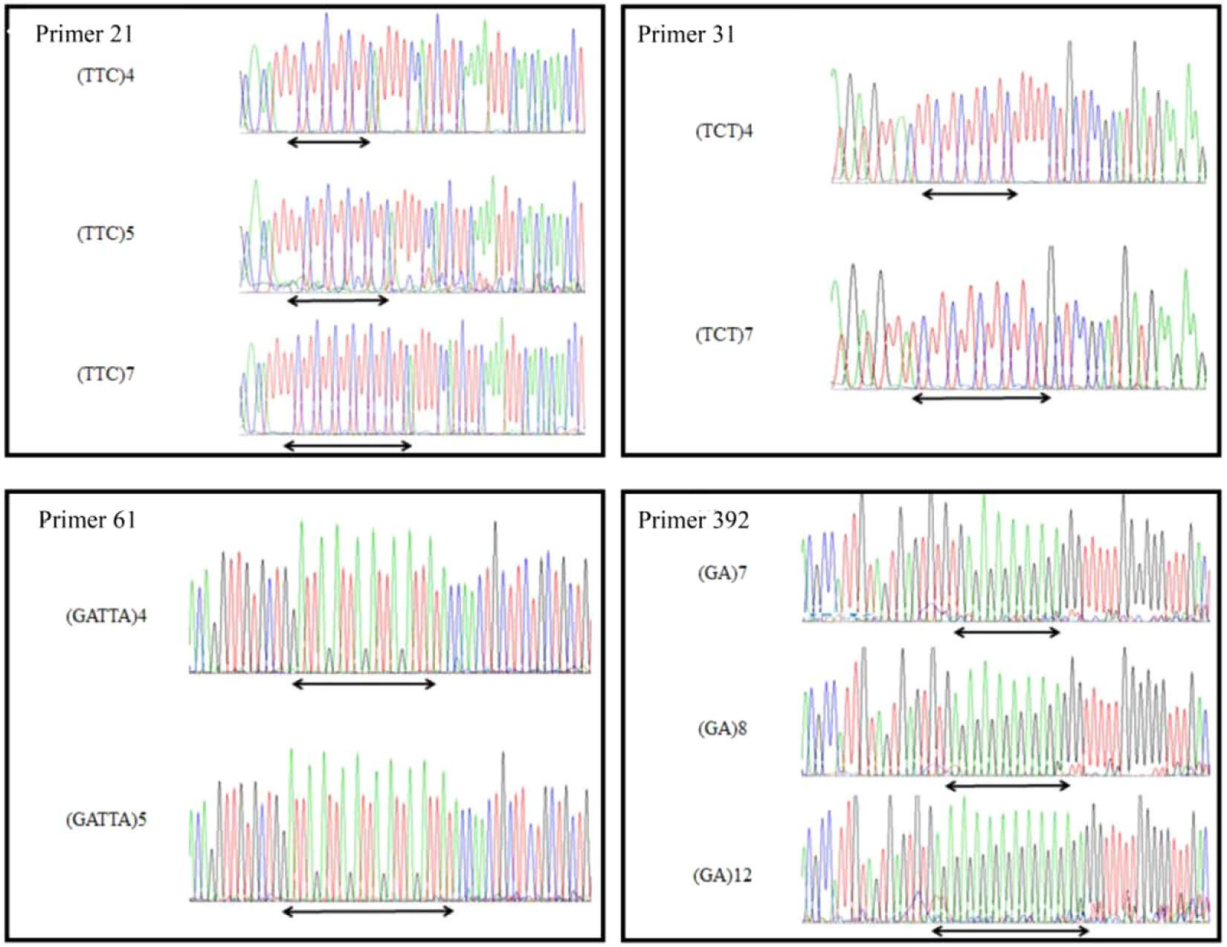
Comparative electropherogram analysis of four EST-SSR loci (primers 21, 31, 61 and 392) among different accessions of *M. albus*. The primer 21 had trinucleotide repeats of (TTC)_4_, (TTC)_5_, and (TTC)_7_. (TCT)_4_, and (TCT)_7_ were obtained using primer 31. Primer 61 can generate tandem repeats of (GATTA)_4_ and (GATTA)_5_. (GA)_7_, (GA)_8_ and (GA)_12_ were obtained by using primer 392.

In the initial screen of 550 EST-SSR primer pairs were selected based on the conditions of base repeat type, fragment length and annealing temperature by using the genomic DNA of fifteen accessions of *M. albus*. A total of 351 pairs of primers generated amplification products, while the remaining 199 pairs of primers failed to detect PCR amplification products at multiple annealing temperatures. For the 351 that amplified, 290 pairs of primers were obtained with clear and well-sized amplified products, while the remaining 61 pairs of primers amplified the PCR product bands size is greater or less than the expected fragment size of the primer. Of the 290 pairs of EST-SSR primers capable of amplifying the expected fragment size of the primers, 206 pairs showed polymorphism, while the remaining 84 primers did not (Table S2). Then, 290 EST-SSR primer pairs were selected for further screening based on their polymorphism using sixty individuals of fifteen *M. albus* accessions. In total, 114 polymorphism primers were selected and used for transferability analysis of the genus *Melilotus* (Fig. 1, Table S3). Furthermore, to verify the accuracy and authenticity of the PCR amplification bands in this study, PCR products of four pairs of primers (21, 31, 61, 392) were selected and sequenced. As shown in Fig. 2, primer 21 had three-base repeats, (TTC)_4_, (TTC)_5_, and (TTC)_7_; (TCT)_4_ and (TCT)_7_ were observed in primer 31; and primer 61 contained tandem repeats of (GATTA)_4_ and (GATTA)_5_. (GA)_7_, (GA)_8_ and (GA)_12_ were obtained using primer 392.

### Transferability of the newly developed EST-SSR markers

Of the 114 primer pairs, 70 successfully amplified sequence from all accessions of 18 species of the genus *Melilotus* and showed high polymorphism (Table 2). A total of 411 alleles were detected using 70 EST-SSR loci in 54 *Melilotus* individuals, ranging from 2 to 11 per locus. Primer 357 yielded the highest number of alleles, and the lowest numbers of alleles were obtained from primers 267 and 383. The observed heterozygosity (*H*_*O*_) ranged from 0.0 to 1.00 with an average of 0.11. The expected heterozygosity (*H*_*E*_) ranged from 0.10 to 0.88 with an average of 0.72. The *PIC* values ranged from 0.10 to 0.87, with an average of 0.69 (Table 2).

**Table 2:** Polymorphism analysis of 70 EST-SSR primers with 18 *Melilotus* species.

Primer code	*N*_*A*_	*H*_*O*_	*H*_*E*_	*PIC*
1	8	0.02	0.83	0.80
3	8	0.11	0.75	0.73
4	4	0.08	0.65	0.60
10	4	0.10	0.72	0.67
11	6	0.00	0.78	0.75
17	3	0.00	0.65	0.58
21	5	0.03	0.76	0.73
22	5	0.25	0.76	0.72
36	5	0.04	0.75	0.71
37	5	0.02	0.71	0.65
39	4	0.08	0.67	0.60
55	5	0.04	0.44	0.42
56	5	0.08	0.71	0.67
61	7	0.24	0.81	0.78
62	7	0.02	0.70	0.67
63	6	0.10	0.78	0.74
66	7	0.11	0.82	0.79
72	7	0.06	0.80	0.77
76	5	0.04	0.73	0.68
79	6	0.04	0.78	0.75
86	5	0.00	0.77	0.74
87	4	0.10	0.67	0.60
89	5	0.07	0.74	0.69
97	4	0.00	0.46	0.42
99	5	0.26	0.76	0.72
102	6	0.11	0.74	0.69
104	5	0.00	0.71	0.66
109	7	0.22	0.82	0.80
112	6	0.06	0.78	0.75
117	5	0.00	0.73	0.68
127	5	0.00	0.74	0.71
132	5	0.02	0.69	0.65
134	6	0.04	0.76	0.72
137	4	0.00	0.67	0.62
140	6	0.00	0.82	0.80
143	3	0.06	0.50	0.42
151	5	0.26	0.70	0.65
170	6	0.04	0.78	0.75
176	3	0.00	0.27	0.24
196	7	0.04	0.79	0.76
213	4	1.00	0.63	0.57
214	5	0.09	0.72	0.68
217	7	0.09	0.80	0.77
219	9	0.36	0.88	0.86
222	7	0.09	0.81	0.79
242	7	0.04	0.76	0.73
252	4	0.00	0.69	0.64
266	7	0.12	0.84	0.82
267	2	0.00	0.14	0.13
278	6	0.39	0.80	0.77
281	9	0.04	0.87	0.86
293	6	0.06	0.78	0.75
294	8	0.15	0.83	0.81
302	9	0.21	0.82	0.80
351	8	0.00	0.81	0.78
357	11	0.07	0.87	0.85
377	6	0.15	0.73	0.70
383	2	0.00	0.10	0.10
392	4	0.93	0.54	0.43
419	9	0.06	0.83	0.81
424	5	0.00	0.73	0.68
433	10	0.13	0.88	0.87
447	7	0.51	0.79	0.77
451	6	0.04	0.77	0.73
461	8	0.20	0.82	0.80
472	8	0.00	0.82	0.79
508	5	0.03	0.72	0.67
545	5	0.26	0.74	0.70
547	8	0.04	0.84	0.82
548	5	0.08	0.73	0.69
Mean	5.87	0.11	0.72	0.69

Note: *N*_*A*_, number of alleles, *H*_*O*_, observed heterozygosity, *H*_*E*_, expected heterozygosity, *PIC*, polymorphic information content.

### Genetic diversity analysis and population structure analysis

As shown in Table 3, the number of polymorphic loci (NPL) for 18 species of *Melilotus* ranged from 1 (*M. sulcatus*) to 136 (*M. segetalis*), and the highest and lowest percentages of polymorphic loci (PPLs) were 33.09% and 0.24%, respectively. The observed number of alleles (na) varied from 1.002 (*M. sulcatus*) to 1.331 (*M. segetalis*), and the effective number of alleles (ne), Nei’s gene diversity (h) and Shannon’s Information index (I) values ranged from 1.002 (*M. sulcatus*) to 1.176 (*M. segetalis*), 0.001 (*M. sulcatus*) to 0.112 (*M. segetalis*) and 0.002 (*M. sulcatus*) to 0.171 (*M. segetalis*), respectively.

**Table 3:** Genetic diversity of 18 *Melilotus* species was detected by 70 EST-SSR markers.

Species	NPL	PPL (%)	na	ne	h	I
*M. albus*	106	25.79%	1.258	1.173	0.1	0.147
*M. altissimus*	30	7.30%	1.073	1.05	0.029	0.042
*M. dentatus*	57	13.87%	1.139	1.093	0.054	0.079
*M. elegans*	20	4.87%	1.049	1.035	0.02	0.029
*M. hirsutus*	47	11.44%	1.114	1.076	0.044	0.065
*M. indicus*	34	8.27%	1.083	1.052	0.031	0.046
*M. infestus*	68	16.55%	1.166	1.109	0.063	0.094
*M. italicus*	21	5.11%	1.051	1.033	0.019	0.029
*M. officinalis*	103	25.06%	1.251	1.16	0.094	0.14
*M. polonicus*	24	5.84%	1.058	1.042	0.024	0.034
*M. segetalis*	136	33.09%	1.331	1.176	0.112	0.171
*M. siculus*	22	5.35%	1.054	1.036	0.021	0.031
*M. speciosus*	15	3.65%	1.037	1.026	0.015	0.021
*M. spicatus*	113	27.49%	1.275	1.166	0.1	0.15
*M. suaveolens*	34	8.27%	1.083	1.058	0.033	0.048
*M. sulcatus*	1	0.24%	1.002	1.002	0.001	0.002
*M. wolgicus*	18	4.38%	1.044	1.03	0.017	0.025
*M. tauricus*	40	9.73%	1.097	1.069	0.039	0.057

Note: NPL, the number of polymorphic loci; PPL, the percentage of polymorphic loci; na, observed number of alleles; ne, effective number of alleles; h, Nei’s (1973) gene diversity; I, Shannon’s Information index.

Using NTSYS-pc.V.2.1 software and the UPGMA method, 58 individual plants of 15 germplasm of *M. albus* were divided into three clusters (Fig. S1). Cluster I contained five germplasms, PI 478773, PI 508617, PI 342796, PI 662296 and PI 478468. Cluster II contained PI 342765, PI 494706, ZXY06P-1732, Zhongxu-1226 and ZXY07P-3150, and the remaining five accessions were clustered in Cluster III. From the results of the cluster analysis, it can be seen that the individual plants of the germplasm were clustered together, and the genetic similarity coefficients ranged from 0.79 to 0.98. The genetic similarity coefficients were higher, which indicated that the genetic relationship between the 15 germplasms was close.

**Figure 3:**
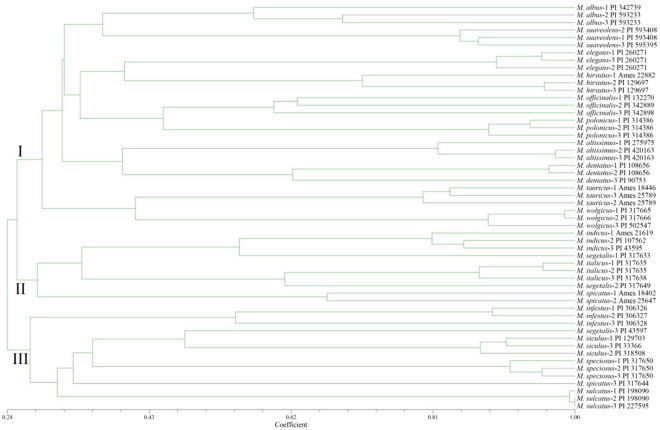
Cluster analysis of 18 species of the *Melilotus* genus based on 70 EST-SSR markers.

According to the UPGMA dendrogram (Fig. 3), 18 *Melilotus* species were classified into three clusters, among which Cluster I contained the 10 species *M. albus*, *M. altissimus*, *M. dentatus*, *M. elegans*, *M. hirsutus*, *M. officinalis*, *M. polonicus*, *M. suaveolens*, *M. tauricus* and *M. wolgicus*. Cluster II and Cluster III contained four species, respectively, except for the germplasm PI 43597 of *M. segetalis* and the germplasm PI 317644 of *M. spicatus*.

**Figure 4:**
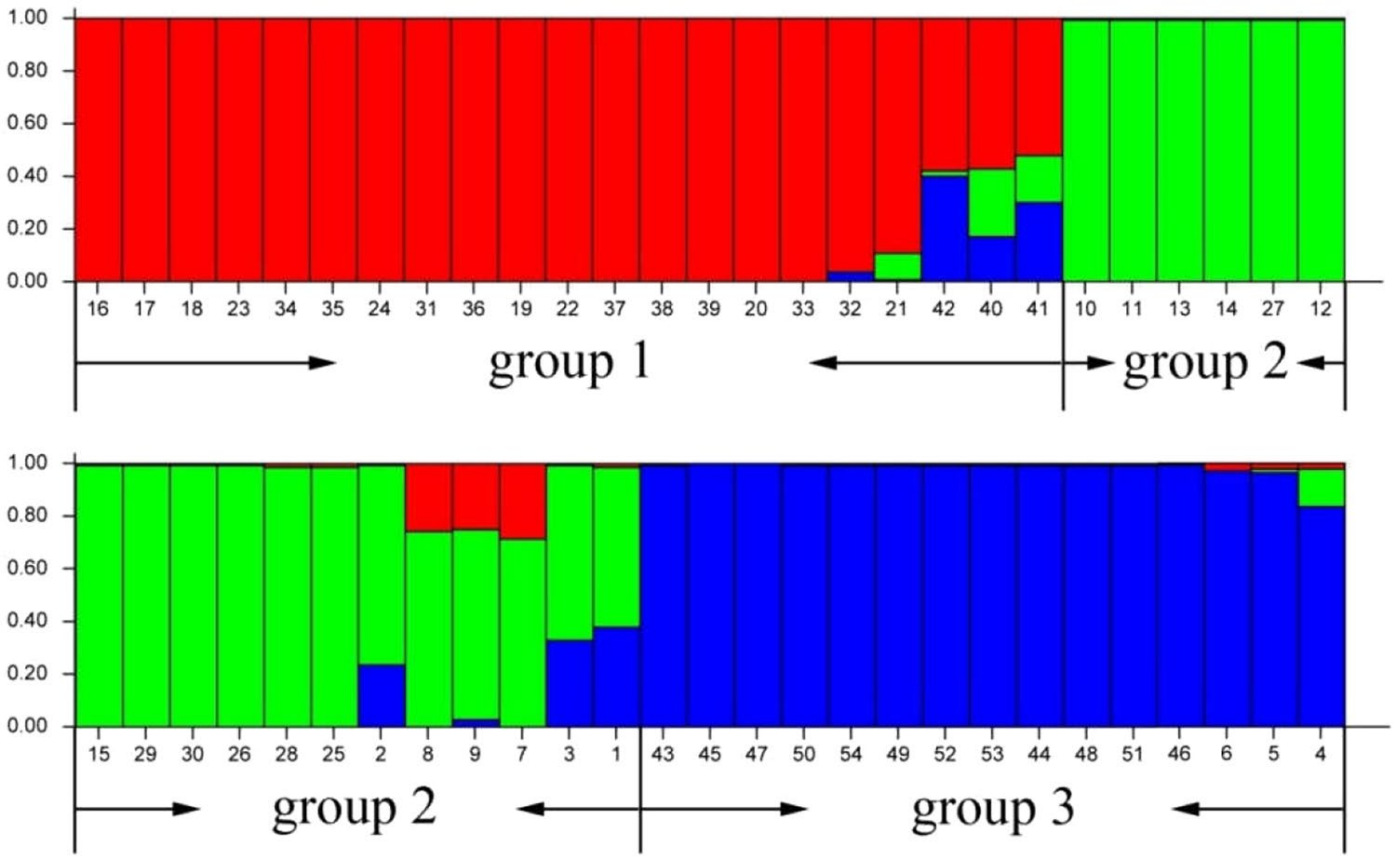
Genetic structure of 54 individuals for 18 *Melilotus* species as inferred by STRUCTURE with the EST-SSR marker data set. Histogram of the STRUCTURE analysis for the model with K = 3 (showing the highest ΔK). The smallest vertical barre presents one individual. The assignment proportion of each individual into Cluster I, Cluster II and Cluster III is shown along the y-axis.

The analysis of the genetic structures of 54 individual plants belonging to 18 *Melilotus* species by using EST-SSR markers was performed with structure software, which was run for K = 2–8. The optimal number of groups was three based on maximum likelihood and delta K (ΔK) values (Fig. 4). Among them, Group I contained 21 individuals belonging to the 7 species *M. indicus*, *M. infestus*, *M. italicus*, *M. segetalis*, *M. siculus*, *M. speciosus* and *M. spicatus*. Group II contained 18 individuals belonging to the 6 species *M. albus*, *M. dentatus*, *M. elegans*, *M. hirsutus*, *M. officinalis* and *M. polonicus*. Group III contained the remaining 15 individuals, which belonged to the 5 species *M. altissimus*, *M. suaveolens*, *M. sulcatus*, *M. tauricus* and *M. wolgicus*.

## Discussion

SSR markers are well known and widely used for genetic diversity analysis, germplasm identification, comparative genetics, phylogenetic relationship, QTL analysis, linkage mapping and marker-assisted selection^26,27^. Therefore, they are useful for studying genetics and for breeding applications to develop SSR markers from the *M. albus* transcriptome. In this study, a total of 19,263 potential EST-SSRs were identified from 104,358 *Melilotus* unigenes, revealing that the abundance of SSRs for *Melilotus* ESTs was higher than those for alfalfa^28^, *M. truncatula* ^29^, and cupuassu tree^30^. In total, 18,182 EST–SSR markers were identified from the 19,263 potential EST-SSRs. The study did not analyse the mononucleotide repeats because it was difficult to distinguish single nucleotide repeats from polyadenylation products and single nucleotide stretch errors produced by sequencing^28^. Here, di- and tri-nucleotide repeats were the most abundant repeats in *Melilotus*, which is same as in most plants in previous studies. Dinucleotide repeats were found to be the most abundant repeat motif in pistachio^21^, tea^31^, rubber tree^32^ and *Neottopterisnidus* ^33^. Tri-nucleotide repeats were the most abundant repeat motif in bread wheat^34^, Siberian wildrye^35^, alfalfa^28^ and castor bean^36^.

Fifteen *M. albus* accessions were used to screen the 550 pairs of primer for PCR amplification, and clear bands were detected with 351 pairs. This was fewer than for Siberian wildrye^35^ but higher than alfalfa^28^ and rubber tree^32^. Among these 351 primer pairs that produced amplification, 290 pairs of primers were obtained with clear and well-sized amplified products, while the remaining 61 primer pairs amplified PCR product bands that were larger or smaller than the expected. These deviations may be due to the large insertions or to variation in the repeat number, lack of specificity or assembly error and the existence of introns^37,38^. However, 114 of those 290 primer pairs were polymorphic among 60 individuals of 15 *M. albus* accessions. In the tested accessions, the percentage of polymorphic loci was higher than the results for Siberian wildrye^35,39^. The high levels of polymorphism observed may be due to the *M. albus* materials selected for screening the primers.

In this study, 114 newly developed EST-SSR markers were used to evaluate the transferability of 18 species of the genus *Melilotus*. In total, 70 of the 114 primer pairs successfully amplified in all species and obtained stable transferability (61.4%), which is higher than the rates obtained in Siberian wildrye (49.11%)^35^ and *Cucumis* (12.7%)^40^. The high rate of transferability of EST-SSRs was may be because all genotypes belonged to the genus *Melilotus*, which means that the EST-SSR markers are derived from transcribed regions that are conserved across species. Additionally, the EST-SSRs have higher transferability than SSRs were from untranscribed regions^41^. Molecular markers are useful for evaluating the genetic diversity in crop species. The genetic diversity estimated by EST-SSR loci was supported by the high values of *N*_*A*_, *H*_*O*_, *H*_*E*_ and *PIC*. In this study, the avenge values of *N*_*A*_, *H*_*O*_, *H*_*E*_ and *PIC* were 5.87, 0.11, 0.72 and 0.69, respectively. These values were lower than those seen in our previous studies in *Melilotus* ^7^. This may be because the expressed sequences, from which EST-SSR are derived, are highly conserved. EST-SSR markers detected a lower rate of polymorphism than the genomic SSRs^42,43^. However, the average values of *H*_*E*_ and *PIC* were higher than in previous studies in *Elymus* ^35^, alfalfa (*M. sativa* L.)^28^ and millet (*Setariaitalica* L.)^44^. However, the value of *H*_*O*_ was low, with an average of 0.11. Therefore, to analyse the genetic diversity and promote genetic breeding programs in future studies, it is necessary to improve the heterozygosity of *Melilotus* accessions^7^. As shown in the UPGMA results, *M. albus*, *M. altissimus*, *M. dentatus*, *M. elegans*, *M. hirsutus*, *M. officinalis*, *M. polonicus*, *M. tauricus*, *M. wolgicus* and *M. suaveolens* were clustered into single group, which is consistent with the results of previous studies^13^. However, in a previous study, *M. dentatus* and *M. tauricus* were not clustered into the same group as the other species^7^. Among the 40 *Melilotus* germplasms, the association between the clustering pattern and the geographical distribution was less significant. The result may be due to the small number of accessions from each geographical location used in this study. The similar results have been reported in alfalfa^45^ and drumstick^46^. These results revealed that genetic distance cannot be the only criterion for genetic differentiation of populations. Moreover, the genetic structure revealed some species showing admixture between group I and group II, while species showed less admixture in group III. Therefore, it is important to use more EST-SSR loci and more individual plants to reveal the relationships among *Melilotus* species in future studies.

In the present study, we developed a large number of EST-SSR markers for *Melilotus* from transcriptome data. A total of 104,358 unigenes were generated, and 19,263 EST-SSRs were identified. For these EST-SSRs, 18,182 primer pairs were successfully designed, providing an important foundation for molecular marker development in *Melilotus*. Of these EST-SSRs, 550 were selected on the basis of base repeat type, fragment length and annealing temperature for further validation. A total of 114 primer pairs detected high polymorphism among 15 accessions of *M. albus*. In addition, 70 showed transferability of 114 polymorphic primer pairs among 18 *Melilotus* species. The results suggest that these 70 primer pairs will be useful in future studies of *Melilotus* population structure, genetic diversity, molecular assisted selection, QTL analysis, and evaluation of germplasm accessions. This study offers a valuable resource for the genetic diversity and molecular-assisted breeding of germplasm resources in the genus *Melilotus*.

## Materials and Methods

### Plant materials and DNA extraction

Seeds from *Melilotus* species were obtained from the National Gene Bank of Forage Germplasm (NGBFG, China) and the National Plant Germplasm System (NPGS, USA) as summarized in Tables 4 and 5. Fifteen accessions of *M. albus* were used to screen EST-SSR markers for polymorphism and to assess genetic diversity (Table 4), and each accession contained four individual plants. Forty accessions of eighteen species in *Melilotus* genus were collected to evaluate the transferability of these newly developed EST-SSR markers to other related species and to analyse genetic diversity among *Melilotus* species (Table 5), and each species contained three individual plants from different accessions. Genomic DNA was extracted from the young leaves using the sodium dodecyl sulfate (SDS) method^47^. The extracted DNA was detected by agarose gel electrophoresis. The samples were diluted with ddH_2_O to 50 ng/μL and stored at −20 °C.

**Table 4:** *M. albus* accessions used for EST-SSR molecular marker validation.

Code	Accession number	Origin	Latitude	Longitude
1	PI 662299	Vienna, Austria	N 48°20′	E 16°33′
2	PI 553075	Poland	N 51°48′	E 19°06′
3	PI 366038	Buenos Aires, Argentina	S 34°35′	W 58°26′
4	PI 478773	Florida, United States	N 27°39′	W 81°30′
5	PI 508617	Santa Fe	S 32°5′	E 1°29′
6	PI 342796	Hungary	N 47°05′	E 19°36′
7	PI 662296	Saskatchewan, Canada	—	—
8	PI 478468	Bolivia	—	—
9	PI 342765	France	N 46°15′	W 2°16′
10	PI 494706	Romania	N 44°12′	E 28°36′
11	ZXY06P-1732	Russian Federation	N 62°	W 9°
12	Zhongxu-1226	—	—	—
13	ZXY07P-3150	—	—	—
14	ZXY05P-983	Russian Federation	—	—
15	HB2009-153	Xinyang, China	N 32°10′	E 114°07′

Note: “—” indicates that the information is unknown.

**Table 5:** Accessions of 18 *Melilotus* species used for analysis of primer transferability.

Species	Accession number	Origin	Latitude	Longitude
*M. albus*	PI 342739	England, United Kingdom	N 52°26′	W 19°06′
	PI 593233	Wisconsin, United States	N 43°40′	W 88°33′
*M. altissimus*	PI 275975	France	N 48°38′	W 4°18′
	PI 420163	France	N 46°13′	E 2°12′
*M. dentatus*	PI 108656	Armenia	N 40°4′	E 45°2′
	PI 90753	China	N 35°51′	E 104°11′
*M. elegans*	PI 260271	Ethiopia, Shewa	N 9°9′	E 37°48′
*M. hirsutus*	Ames 22882	Russian Federation	—	—
	PI 129697	Sweden	N 60°7′	E 18°38′
*M. indicus*	Ames 21619	Nebraska, United States	N 41°29′	W 99°54′
	PI 107562	Uzbekistan	N 41°23′	E 69°4′
	PI 43595	—	—	—
*M. infestus*	PI 306326	Algeria	N 27°13′	E 2°29′
	PI 306327	Italy	N 41°52′	E12°34′
	PI 306328	Hungary	N 47°9′	E 19°30′
*M. italicus*	PI 317635	Czechoslovakia	N 14°28′	E 121°2′
	PI 317638	Israel	N 31°2′	E 34°51′
*M. officinalis*	PI 132270	Romania	—	—
	PI 342889	Germany	—	—
	PI 342898	France	—	—
*M. polonicus*	PI 314386	Former Soviet Union	N 45°5′	E 41°50′
*M. segetalis*	PI 317633	Algeria	N 27°13′	E 2°29′
	PI 317649	Czechoslovakia	N 48°2′	E 18°22′
	PI 43597	—	—	—
*M. siculus*	PI 129703	Malta	N 35°56′	E 14°22′
	PI 318508	Greece	N 39°4′	E 21°49′
	PI 33366	Former Soviet Union	—	—
*M. speciosus*	PI 317650	Manitoba, Canada	N 53°45′	W 98°48′
*M. spicatus*	Ames 18402	Nebraska, United States	N 41°29′	W 99°54′
	Ames 25647	Krym, Ukraine	N 44°24′	E 33°49′
	PI 317644	Algeria	N 27°13′	E 2°29′
*M. suaveolens*	PI 593408	South Dakota, United States	N 43°58′	W 99°54′
	PI 595395	Iowa, United States	N 41°52′	W 93°5′
*M. sulcatus*	PI 198090	Morocco	N 31°47′	W 7°5′
	PI 227595	Tunisia	N 33°53′	E 9°32′
*M. tauricus*	Ames 18446	Nebraska, United States	N 41°29′	W 99°54′
	Ames 25789	Krym, Ukraine	N 44°24′	E 33°49′
*M. wolgicus*	PI 317665	Denmark	N 56°15′	E 9°30′
	PI 317666	Czechoslovakia	N 48°2′	E 18°22′
	PI 502547	Russian Federation	N 50°45′	E 49°19′

Note: “—” indicated that the information is unknown.

### Detection of the EST-SSR markers and primer design

SSRs were detected in the assembled unigenes using the Simple Sequence Repeat Identification Tool program (MicroSatellite), and the SSRs were considered to contain mono-, di-, tri-, tetra-, penta-, and hexa-nucleotides with minimum repeat numbers of ten, six, five, five and five, respectively. The EST-SSR primers were designed using BatchPrimer3, and the designed EST-SSR primers were synthesized by Shanghai Sangon Biological Engineering Technology (Shanghai, China).

### Primer selection and PCR conditions

A total of 550 primer pairs were selected according to the conditions of base repeat type (except for single bases), fragment length (150–200 bp) and annealing temperature (55–60 °C). PCR and electrophoresis were performed to screen EST-SSR primer pairs for polymorphism using *Melilotus* species. PCR amplifications were performed in a final volume of 10 μL containing 1 μL genomic DNA (50 ng/μL), 4.9 μL 2 × Reaction Mix (dNTPs at 500 μM each, 20 mM Tris–HCl, 100 mM KCl, 3 mM MgCl_2_), 0.1 μL 2.5 U/μL Golden DNA Polymerase, 1.0 μL of each primer (4  μM each) and 2.0 μL double distilled water. PCR cycling conditions were 3 min at 94 °C, 35 cycles of 30 s at 94 °C, 30 s at the annealing temperature, 30 s at 72 °C and a final extension step of 7 min at 72 °C. The PCR products were subjected to electrophoresis on 6.0% non-denaturing polyacrylamide gels and stained using silver dye. In addition, the DL500 DNA marker was used to determine the sizes of the PCR products.

### Sequencing of PCR amplification products

To verify the accuracy and authenticity of the PCR amplification products, we selected some PCR amplification products for sequencing by Shanghai Sangon Biological Engineering Technology (Shanghai, China). PCR products should be selected according to the presence of single bands and high amplification efficiency.

### Data analysis

The number of alleles of each EST-SSR locus was calculated based on presence (1) or absence (0). The observed heterozygosity (*H*_*O*_), expected heterozygosity (*H*_*E*_), and polymorphic information content (*PIC*) were calculated as previously reported^48^. The program POPGENE 32^49^ was used to calculate the number of polymorphic loci (NPL), the percentage of polymorphic loci (PPL), the observed number of alleles (na), the effective number of alleles (ne), Nei’s gene diversity (h) and Shannon’s Information index (I) values. With the help of SAHN-clustering in NTSYSpc-v.2.1 software, Nei’s unbiased genetic distance and UPGMA were used for cluster analysis to generate a dendrogram^50^. The model-based approach implemented was used to subdivide the individuals into different subgroups in the program STRUCTURE 2.3^51^.

### Data availability

The RNA-seq data supporting the results of this article are available at NCBI under BioProject with accession PRJNA331091.

## Electronic supplementary material

Supplementary Information

Figure S1

Table S1

Table S2

Table S3
