# Supplementary material for: Cross-species transferability of EST-SSR markers developed from the transcriptome of Melilotus and their application to population genetics research
Source: Sci Rep. 2017 Dec 20;7:17959. doi: 10.1038/s41598-017-18049-8 (PMC5738344; doi:10.1038/s41598-017-18049-8)
Supplement: Supplementary file 1 — Supplementary Information [file 41598_2017_18049_MOESM1_ESM.pdf]

Cross-species transferability of EST-SSR markers developed from the transcriptome of *Melilotus* and their application to population genetics research

Zhuanzhuan Yan, Fan Wu, Kai Luo, Yufeng Zhao, Qi Yan, Yufei Zhang, Yanrong Wang, Jiyu Zhang*

State Key Laboratory of Grassland Agro-ecosystems, College of Pastoral Agriculture Science and Technology, Lanzhou University, Lanzhou 730020, China

*Corresponding author: Jiyu Zhang

E-mail: zhangjy@lzu.edu.cn

Supplemental Figure legends

Supplemental Figure 1 Dendrogram of 15 *M. albus* accessions based on 114 EST-SSR markers.

Supplemental Table legends

Supplemental Table 1 Summary of the transcriptome sequencing in *M. albus*

Supplemental Table 2 Summary of 550 EST-SSR primers by PCR amplification

Supplemental Table 3 Polymorphism analysis of 114 novel EST-SSR markers in *Melilotus*

N_A, number of alleles, H_O, observed heterozygosity, H_E, expected heterozygosity, *PIC*, polymorphic information content.
