# Supplementary figures and images for: Cross-species transferability of EST-SSR markers developed from the transcriptome of Melilotus and their application to population genetics research
Source: Sci Rep. 2017 Dec 20;7:17959. doi: 10.1038/s41598-017-18049-8 (PMC5738344; doi:10.1038/s41598-017-18049-8)

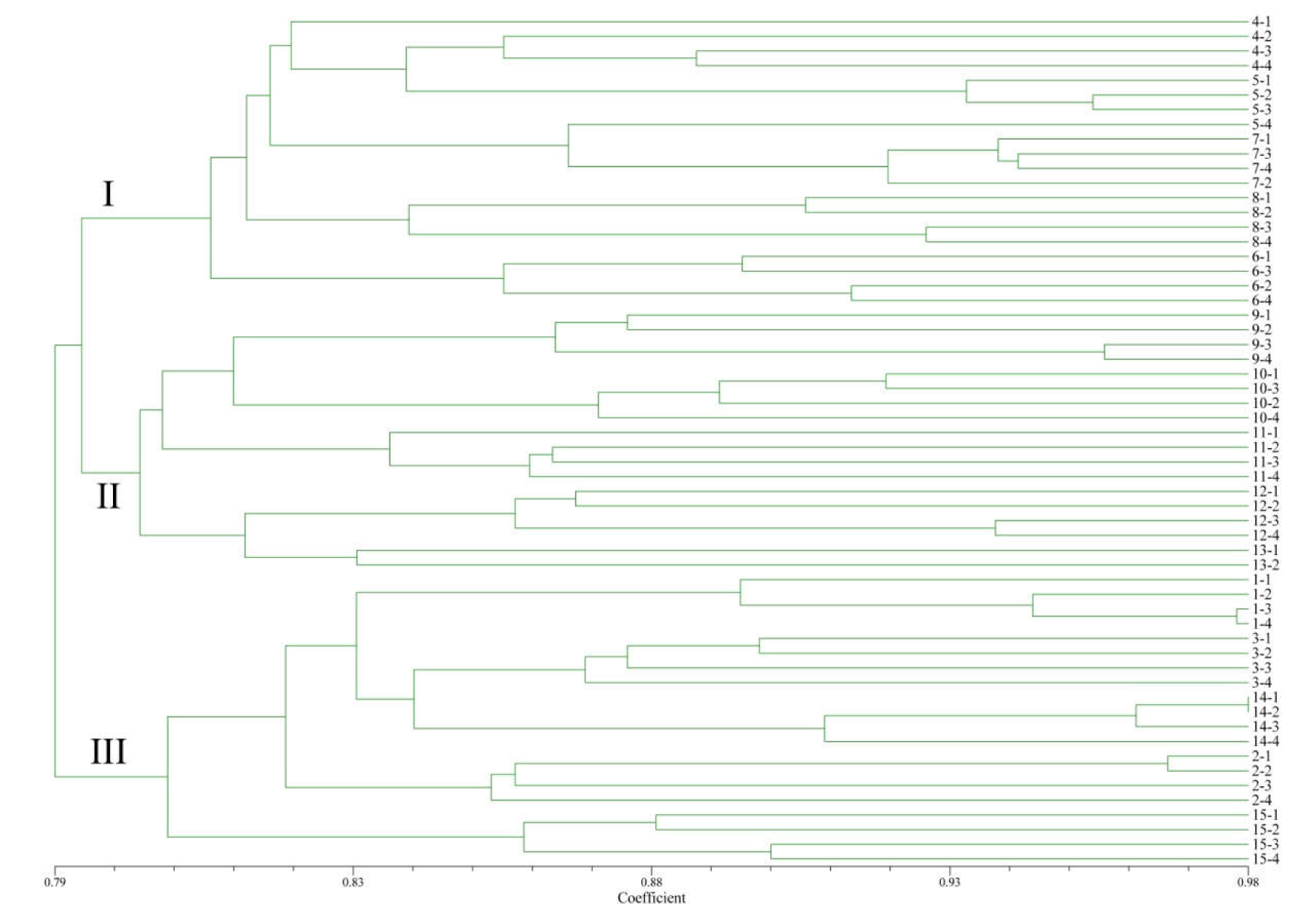

Supplement: Supplementary file 2 — Figure S1 [file 41598_2017_18049_MOESM2_ESM.tif]
