# Supplementary material for: Cross-species transferability of EST-SSR markers developed from the transcriptome of Melilotus and their application to population genetics research
Source: Sci Rep. 2017 Dec 20;7:17959. doi: 10.1038/s41598-017-18049-8 (PMC5738344; doi:10.1038/s41598-017-18049-8)
Supplement: Supplementary file 3 — Table S1 [file 41598_2017_18049_MOESM3_ESM.doc]

**Supplemental Table 1 Summary of the transcriptome sequencing in *M. albus***

| Items | Number | | | | |
| --- | --- | --- | --- | --- | --- |
|  | N46 | N47 | N48 | N49 | RPh |
| Raw Reads | 32939751 | 31176000 | 32518646 | 31470600 | 35446843 |
| Clean Reads | 30532020 | 28785103 | 30067146 | 29041739 | 32634517 |
| Total transcripts | 154458 | | | | |
| Total unigenes | 104358 | | | | |
| Total number of identified SSRs | 19263 | | | | |
| Number of SSR containing sequences | 15260 | | | | |
| Number of sequences containing more than one SSR | 3063 | | | | |
| Number of SSRs present in compound formation | 1081 | | | | |
