# Supplementary material for: Cross-species transferability of EST-SSR markers developed from the transcriptome of Melilotus and their application to population genetics research
Source: Sci Rep. 2017 Dec 20;7:17959. doi: 10.1038/s41598-017-18049-8 (PMC5738344; doi:10.1038/s41598-017-18049-8)
Supplement: Supplementary file 4 — Table S2 [file 41598_2017_18049_MOESM4_ESM.doc]

**Supplemental Table 2 Summary of 550 EST-SSR primers by PCR amplification**

| Items | Number | Percentage (%) |
| --- | --- | --- |
| Not amplify | 199 | 36.2 |
| Successfully amplified | 351 | 63.8 |
| Not expected size | 61 | 11.1 |
| Expected size | 290 | 52.7 |
| Polymorphic | 114 | 20.7 |
| Monomorphic | 182 | 33.1 |
| Total | 550 | 100 |
