# Supplementary material for: Cross-species transferability of EST-SSR markers developed from the transcriptome of Melilotus and their application to population genetics research
Source: Sci Rep. 2017 Dec 20;7:17959. doi: 10.1038/s41598-017-18049-8 (PMC5738344; doi:10.1038/s41598-017-18049-8)
Supplement: Supplementary file 5 — Table S3 [file 41598_2017_18049_MOESM5_ESM.doc]

**Supplemental Table 3 Polymorphism analysis of 114 novel EST-SSR markers in *Melilotus***

| Primer code | Forward primer (5'-3') | Reverse primer (5'-3') | Tm (°C) | Repeats | *N_A_* | *H_O_* | *H_E_* | *PIC* | Transferability |
| --- | --- | --- | --- | --- | --- | --- | --- | --- | --- |
| 1 | F: GGTGTCACTGATCCTCCAGC | R: TGGATGGAAATTGGGGTGAGG | 60.11 | (TCT)5 | 8 | 0.27 | 0.83 | 0.81 | Yes |
| 3 | ACGAGGATCCATCTAGGGTTCA | TGGTCACAAACATTAGACAACGG | 60.09 | (GAT)5 | 14 | 0.28 | 0.87 | 0.86 | Yes |
| 4 | TGCTGTTGATGATGCTTAACACT | GGCGGTTCAAAATCCGGTTC | 58.93 | (TGA)6 | 10 | 0.27 | 0.85 | 0.84 | Yes |
| 10 | ACGCACCTATCTTCCGTTCC | ACTTCTGCAATCAATCTCCAACA | 59.83 | (GTTT)5 | 10 | 0.30 | 0.84 | 0.82 | Yes |
| 11 | ACCAACAGTTACAAGAAGTGAGT | TTTGTGAGGGTTGCGGTGAT | 57.90 | (AACT)5 | 6 | 0.29 | 0.76 | 0.72 | Yes |
| 14 | TGAGGATGGAGATTCATTCATCCA | TACACACAGGGCCCATCAAC | 59.34 | (CT)6 | 7 | 0.07 | 0.81 | 0.78 | No |
| 17 | TGGAAGTGGATGACATGCCC | AATCACACCTGGGTTGGCAA | 60.03 | (TGA)7 | 10 | 0.28 | 0.86 | 0.84 | Yes |
| 20 | GAAAAGCGACGACGATGACG | GGTCGTCCTCTGTCCACTTG | 59.98 | (GAT)5 | 7 | 0.18 | 0.74 | 0.71 | No |
| 21 | GGAAGAGAGAGAAACAGCGCA | GTTCCGGCGAAGAGAGAGAA | 60.34 | (TTC)5 | 13 | 0.38 | 0.85 | 0.84 | Yes |
| 22 | ACCTTTGTCCCCTCACAATCA | AATCTCTGCAGCTTGGCCAT | 59.22 | (AC)8 | 12 | 1.00 | 0.83 | 0.81 | Yes |
| 31 | CGTGGTCGTGGAAACCCTAA | AGAAAGTGTCTAGCGGTGGC | 59.97 | (TCT)5 | 11 | 0.32 | 0.87 | 0.85 | No |
| 36 | AGAAAACACCAGCACCACCA | GCCAACACCGTTCACACTTC | 60.03 | (GAA)5 | 7 | 0.27 | 0.79 | 0.77 | Yes |
| 37 | ATCACTTATAGTCCACAGAAGTGT | GCTTCGTTTAGGCAGCCAAG | 57.14 | (AT)8 | 12 | 0.50 | 0.89 | 0.88 | Yes |
| 39 | TGACTTTGTTTCGCTGTCGC | TCCAACAGAGCCTCAACCAC | 59.70 | (AGT)5 | 10 | 0.25 | 0.83 | 0.81 | Yes |
| 47 | GTTGAGCAGCCCACCTACTT | ACAAGCTTGTCTAACAGCACCT | 59.96 | (TTC)5 | 14 | 0.33 | 0.89 | 0.88 | No |
| 55 | GGAGCCCAGTTCAGAATTCCA | CACCACCACCCTTCTGCATA | 60.00 | (TCC)5 | 8 | 0.19 | 0.83 | 0.81 | Yes |
| 56 | GTACAAGAGCCCTCTCGTCG | TGAAGAGGAAAGTCGGTGGC | 59.90 | (CTT)5 | 11 | 0.28 | 0.86 | 0.85 | Yes |
| 61 | TGACTTCCTTCCCACTTGCC | CACAGCAGCAGAAGCAGTTG | 59.89 | (GATTA)5 | 16 | 0.38 | 0.92 | 0.92 | Yes |
| 62 | GGAGAACACATCACTCTCCCA | ACTGACACCTTGCTGGATCG | 59.10 | (AAAC)5 | 14 | 0.38 | 0.92 | 0.91 | Yes |
| 63 | ACACAAGCAATCGCCAACTT | ATTGGTTGCCTCAGACAGGG | 58.97 | (ATTA)5 | 14 | 0.32 | 0.92 | 0.91 | Yes |

**Continue Supplemental Table 3 Polymorphism analysis of 114 novel EST-SSR markers in *Melilotus***

| Primer code | Forward primer (5'-3') | Reverse primer (5'-3') | Tm (°C) | Repeats | *N_A_* | *H_O_* | *H_E_* | *PIC* | Transferability |
| --- | --- | --- | --- | --- | --- | --- | --- | --- | --- |
| 64 | ACCCCTCATACAGCAGTTGA | TGGAGGCCAACTTCCGTTAG | 58.34 | (AT)6 | 12 | 0.08 | 0.89 | 0.88 | No |
| 66 | ATGTCGAATCGTTACGGCCA | TTGCTACCGGAACCAGAACC | 59.83 | (CAA)5 | 11 | 0.23 | 0.84 | 0.83 | Yes |
| 72 | GTTTTGAGTCCAACCACCACA | CCAACCGTGCCTGTTTCAAG | 58.90 | (CCA)5 | 14 | 0.37 | 0.88 | 0.87 | Yes |
| 76 | GGAGAAGAGTGAAAGTGAAGCC | AGCACCTCAGCTGATGAACC | 58.93 | (GCA)6 | 7 | 0.27 | 0.83 | 0.81 | Yes |
| 79 | ATCTCAAAATCTGCCGCCCA | GTGGTTGAAGGGGAGGTAGC | 60.03 | (CTC)5 | 10 | 0.27 | 0.87 | 0.86 | Yes |
| 81 | ACAGAAGCCACAGGTCTACTC | CGGGATGTTGTGTTGTGCAG | 59.10 | (CT)7 | 10 | 0.00 | 0.83 | 0.81 | No |
| 86 | CGTGAGTCGCCGGATAATGT | ACGCGGAGACAAAGAGAGAG | 60.25 | (AAC)6 | 18 | 0.33 | 0.92 | 0.91 | Yes |
| 87 | ACGAAGACAGTAACATGGCCA | GCGCTGGTTTTGTCATTGGA | 59.65 | (AAC)5 | 12 | 0.20 | 0.86 | 0.84 | Yes |
| 89 | TCCAAACTCAAACAAGATGAACCA | TGTGGAGAGGGTTTCATCAGG | 59.05 | (GAT)5 | 11 | 0.25 | 0.87 | 0.85 | Yes |
| 97 | AATGCACATACGCCGTCTCA | CGGTGGTGGAGGAGTAGGTA | 60.11 | (CGC)5 | 11 | 0.15 | 0.86 | 0.84 | Yes |
| 99 | GGCTTCAGAAACATTGCGGG | GTTGGTCTCGACGCAACAAC | 60.11 | (TTG)5 | 10 | 0.12 | 0.85 | 0.84 | Yes |
| 102 | GGGTACGAGGTGTGGTAGGA | GGGTTTGGGTAAGCTTCGGA | 60.32 | (AGA)5 | 16 | 0.18 | 0.90 | 0.89 | Yes |
| 104 | ACCCACTTGACCTTTTGGCA | CCATGCTTTGTCGGCCATTT | 60.03 | (AAGA)5 | 13 | 0.27 | 0.89 | 0.88 | Yes |
| 109 | GTCAATTCCGTTGTCGCCAG | GTCACGCCCCTTTCGTTTTT | 59.83 | (ATC)6 | 15 | 0.23 | 0.88 | 0.87 | Yes |
| 112 | TGAGCATCTTCGTGCTGTGT | TCACCAAATGAATCGAGTTAGGT | 59.97 | (AGA)6 | 11 | 0.32 | 0.87 | 0.86 | Yes |
| 117 | TTTGGAACCATAAAAGTCTCCTTATT | TGGATGGAGAGTGAGAGGGG | 57.05 | (TTC)5 | 10 | 0.17 | 0.86 | 0.84 | Yes |
| 122 | CCAACCACCAATGTTCCTTGT | GGGACCTATGCTCCTATTGCA | 58.96 | (ATT)5 | 18 | 0.29 | 0.93 | 0.92 | No |
| 127 | GGGAAGAGGGAATGGAGCAC | TCGCCATGTCTCTGTCAACA | 60.11 | (GAGT)5 | 13 | 0.25 | 0.91 | 0.90 | Yes |
| 132 | GAGGAGAAACGGAGGCGTAG | TGGACTTGAATCCGACGGTG | 59.90 | (GAA)5 | 14 | 0.23 | 0.87 | 0.86 | Yes |
| 133 | ATATGGTTGCCGGTGGGAAG | CACCACAACACCAACTCCCT | 60.11 | (TTC)6 | 12 | 0.17 | 0.88 | 0.87 | No |

**Continue Supplemental Table 3 Polymorphism analysis of 114 novel EST-SSR markers in *Melilotus***

| Primer code | Forward primer (5'-3') | Reverse primer (5'-3') | Tm (°C) | Repeats | *N_A_* | *H_O_* | *H_E_* | *PIC* | Transferability |
| --- | --- | --- | --- | --- | --- | --- | --- | --- | --- |
| 134 | TCGCCAATTGCCATCCTTCT | AAAGTTAGAGCCCCCATGCC | 60.03 | (CAA)5 | 14 | 0.25 | 0.84 | 0.83 | Yes |
| 137 | TTCCACTCCAAACCCACCAC | TGTTGTCGAGGTCCCAAAGG | 60.11 | (CAA)5 | 12 | 0.25 | 0.89 | 0.88 | Yes |
| 139 | GAGAGGAAGAAGAGGTGGCG | CCACTCACTCTCCTACACAACA | 59.83 | (GAA)5 | 12 | 0.25 | 0.88 | 0.86 | No |
| 140 | ACGGTCGAAGTAGTCCGGTA | GTGGTGGTGATGGTGATGGT | 60.04 | (CTC)5 | 12 | 0.32 | 0.87 | 0.85 | Yes |
| 143 | GACACTGCCTTTTCCGGAGA | GGTTAAGTGAAACGGCGCTG | 59.97 | (CAT)5 | 5 | 0.05 | 0.64 | 0.58 | Yes |
| 151 | AAAACCGAGCTGCAAAGTGC | AACAGGTCTGCGATGGATGG | 60.25 | (AGA)5 | 10 | 0.53 | 0.83 | 0.81 | Yes |
| 167 | TCCAACCACGTACTCCAAGC | ACCAATCAACTGAGATATTCATTGTGG | 59.97 | (CAT)5 | 12 | 0.16 | 0.89 | 0.88 | No |
| 170 | GTGAGACCAGGTAACACCGG | TTTCCGCTTAACCCAAACGC | 60.04 | (GAA)5 | 10 | 0.12 | 0.83 | 0.81 | Yes |
| 175 | CACGTCCAGTCAGGCACTAA | ACATGCAACTGTATTCTAGGCCT | 59.68 | (TTG)5 | 7 | 0.07 | 0.76 | 0.72 | No |
| 176 | TGCAACAAAGTTTGCATCATTGG | ACTGTCACAACACTCTTGGAGA | 59.44 | (AC)6 | 11 | 0.37 | 0.77 | 0.75 | Yes |
| 196 | GAAATTCGGGAGGCCGAGAA | TGTCTGAACTCAAACACCATTTTCA | 60.11 | (TGT)5 | 6 | 0.09 | 0.80 | 0.77 | Yes |
| 199 | TGGAAACGGAGGGAGAAACC | TTTGGGGGAATTGACGGAGG | 59.60 | (ACC)7 | 8 | 0.17 | 0.83 | 0.81 | No |
| 200 | CACCAGCAGAAGAGGAAGAGG | GTGCCATTGCGGTTGAATGT | 60.07 | (TCC)7 | 15 | 0.27 | 0.90 | 0.89 | No |
| 201 | AAACACTTGGCCGGCCTTAT | TGCATGTTAACTAGCTTGGCA | 60.25 | (TATG)5 | 9 | 0.04 | 0.86 | 0.84 | No |
| 212 | GTGAAAGTGGGTTGGGTCCT | GGGAGGCCAGACTCAAGAAC | 59.82 | (AGAGG)5 | 12 | 0.22 | 0.83 | 0.82 | No |
| 213 | GAAAAACCAAAACGCGCCAC | TCGGAGGATAGGGAAGGGTC | 59.70 | (CAT)5 | 7 | 0.97 | 0.70 | 0.67 | Yes |
| 214 | ACGGAGGAGTGGAGTCTTGT | ATTGGAGGCATTGGGGATGG | 60.18 | (CACT)5 | 8 | 0.27 | 0.80 | 0.77 | Yes |
| 217 | ACTTCTCGTGAATGCACAGT | TTTTCTTTCTCATGCTTTGCTAGT | 57.47 | (AC)8 | 8 | 0.12 | 0.82 | 0.80 | Yes |
| 218 | CGGTTCGACTCCAACTCCAA | CATGGGTCTTGGTTTGCGTG | 59.97 | (TTG)5 | 12 | 0.14 | 0.88 | 0.86 | No |
| 219 | TCGGCTGCTTTGTCTCTCTT | GGACGGAACATGGAGGAGTG | 59.32 | (TC)7 | 13 | 0.15 | 0.85 | 0.84 | Yes |

**Continue Supplemental Table 3 Polymorphism analysis of 114 novel EST-SSR markers in *Melilotus***

| Primer code | Forward primer (5'-3') | Reverse primer (5'-3') | Tm (°C) | Repeats | *N_A_* | *H_O_* | *H_E_* | *PIC* | Transferability |
| --- | --- | --- | --- | --- | --- | --- | --- | --- | --- |
| 222 | TCTAATCCGTTTGCGCCGTA | TCGAAGAGTTGGATGCGCTT | 59.83 | (CCT)5 | 8 | 0.27 | 0.77 | 0.75 | Yes |
| 233 | GTGTGTGAAAATGGCGGTTCT | CAGCGTTCACTTTGCTTCCC | 59.66 | (AAC)5 | 13 | 0.23 | 0.86 | 0.85 | No |
| 242 | TGGATCCGTTTTTCTTCCTCTCA | GAAGAAGTTCTGCGATCGGC | 59.67 | (CTT)5 | 8 | 0.10 | 0.77 | 0.74 | Yes |
| 252 | AGCAGTTGAGCCGTGAATCA | TGTTTTGTATCCCGGGGCAG | 59.97 | (ATAG)5 | 14 | 0.38 | 0.90 | 0.89 | Yes |
| 259 | CATTGCCCTCACGCGTTAAA | CGGAGGAAGTACTAATTTACCTTCG | 59.48 | (AATT)5 | 5 | 0.02 | 0.73 | 0.68 | No |
| 266 | CGGTTATGCTCTTTTCAAGGGT | CGACGAGGCCTTGAATTCCT | 58.92 | (CTT)5 | 10 | 0.19 | 0.87 | 0.86 | Yes |
| 267 | GGGCGGTAGAGGAGGTAGAA | AAGCTCAACTCTACGGTGCC | 60.11 | (GAG)5 | 2 | 0.00 | 0.18 | 0.16 | Yes |
| 278 | GAGGGTCTTTTCCGTTCGGT | CTCCCAATCCCAAAGCCCAT | 59.97 | (AAC)6 | 10 | 0.85 | 0.86 | 0.85 | Yes |
| 281 | GGCGAGAATTGTGTGACACG | CTGGAAATCCACGTGGCTGA | 59.84 | (GAAT)5 | 9 | 0.17 | 0.82 | 0.80 | Yes |
| 291 | TCACAGCCTCTCCACCAAAC | AAGCAGCCATAGTGTCGTGA | 59.89 | (TTC)5 | 8 | 0.30 | 0.83 | 0.81 | No |
| 293 | AACTCAAAGGACGGTGGGTG | AATGGTCCTAGCCCACGTTG | 60.18 | (ACA)5 | 9 | 0.09 | 0.75 | 0.72 | Yes |
| 294 | GCAGTTACCAGAAAAGCGGC | TCTCTTCGCGTGAGTGTGAA | 60.11 | (AAAC)5 | 5 | 0.07 | 0.59 | 0.53 | Yes |
| 300 | ACCAGTTAGTTGCTGCTGGT | GGTATGTTTGGTAAGCACAGTGA | 59.53 | (CA)6 | 7 | 0.28 | 0.80 | 0.77 | No |
| 302 | AGAGTCCCACGTTGTTGTAGT | GCAGCTTGCAAGTTTCTGTCA | 58.96 | (CA)11 | 10 | 0.00 | 0.86 | 0.85 | Yes |
| 308 | TCACCCCTCTCACTCTCACA | CAGGGAAAACTGGCCTGGTA | 59.52 | (ATAC)5  (AT)9 | 13 | 0.61 | 0.90 | 0.89 | No |
| 314 | GTCAGACTCCTCCTTCCCCT | CCCTAACCCTACATGTCGGC | 59.96 | (TCC)5 | 3 | 0.93 | 0.51 | 0.39 | No |
| 316 | CTTCCACCACCAATCCCTCC | GGTCGCCGGAGAGTTGTAAT | 60.03 | (ACC)5 | 11 | 0.18 | 0.86 | 0.85 | No |
| 319 | ATGTTGATTAGGCGGCGGAA | GCTGAGTTGTCACGCACTTG | 60.11 | (ATC)5 | 4 | 0.13 | 0.38 | 0.35 | No |
| 320 | TGGCAGTAGCACGTGATGTT | TGTTTGGGTGAGGAGAGTGA | 59.97 | (TTC)6 | 12 | 0.17 | 0.86 | 0.84 | No |

**Continue Supplemental Table 3 Polymorphism analysis of 114 novel EST-SSR markers in *Melilotus***

| Primer code | Forward primer (5'-3') | Reverse primer (5'-3') | Tm (°C) | Repeats | *N_A_* | *H_O_* | *H_E_* | *PIC* | Transferability |
| --- | --- | --- | --- | --- | --- | --- | --- | --- | --- |
| 326 | GATGACTCCCGCGATAGGTG | AGAAGAGGAGGAGGTGGGAG | 60.04 | (TCC)5 | 9 | 0.28 | 0.80 | 0.77 | No |
| 328 | TCTTCTCTCTTTTTCCAGCGT | GGGGAAAAAGGGGTTGAGCT | 57.25 | (ACC)5 | 10 | 0.45 | 0.82 | 0.80 | No |
| 349 | AGCAGGAAAATTGGAGGCCT | TCCTCTTGGTTTGCGTCTGA | 59.59 | (CAG)6 | 14 | 0.40 | 0.91 | 0.90 | No |
| 351 | CCAACAGCAACCAGAGCAAC | GACGATGAAGCGGACGTTTG | 59.97 | (GCA)6 | 7 | 0.17 | 0.77 | 0.74 | Yes |
| 357 | GCGGATTGGGAGAGAGAAGG | GGGTATGGGTGGAGGAGAGT | 59.89 | (TATT)5 | 11 | 0.34 | 0.86 | 0.84 | Yes |
| 377 | TGGAGGACGTAGGTTCAAGTG | ACTGCTAGGCACCAAGTCAA | 59.38 | (TG)6 | 9 | 0.22 | 0.85 | 0.83 | Yes |
| 383 | ATGGCCACCCAGAATGTTGT | CCGTCAAGAATTGCCACAGC | 59.89 | (TTC)5 | 8 | 0.25 | 0.77 | 0.75 | Yes |
| 391 | AGAAGAATCAGGTGCGGTGG | GCTCTGTGTTGAGGAGGGTC | 60.04 | (GATC)5 | 12 | 0.28 | 0.88 | 0.87 | No |
| 392 | GGGATGTGAGAGGGGAGAGT | CTCCTCGAACCTTGACCTGG | 60.03 | (GA)7 | 8 | 0.18 | 0.75 | 0.71 | Yes |
| 393 | CGTCGGTTAGGACTTGTGCT | TTCCTCCTCTTCGGCTGAGA | 60.04 | (TGG)6 | 8 | 0.18 | 0.81 | 0.79 | No |
| 396 | CAACAAATTGGTGTTAGGCAGC | GAGTCCGACAAATGGAGCCA | 58.94 | (TA)6 | 13 | 0.52 | 0.89 | 0.88 | No |
| 397 | AGAATTAAAAGAACGCGCGC | TCCCGGGATATGGATTTGCG | 58.12 | (CA)6 | 10 | 0.35 | 0.86 | 0.85 | No |
| 402 | CCTGAGAGGATGAGGGGTGA | CTCCTGTGATGGACCGCTTT | 60.03 | (GA)8 | 11 | 0.23 | 0.85 | 0.84 | No |
| 406 | TGCCTCAGTTGAACGAACGA | GTGCGGTGCCCATTTGAATT | 59.90 | (AAT)6 | 9 | 0.13 | 0.82 | 0.80 | No |
| 409 | TGAGGGAGAAAAGGGTGAGG | AGTTTTGGTGAGAAAGGGGCA | 58.64 | (GA)8 | 13 | 0.60 | 0.90 | 0.89 | No |
| 411 | TGGTGCATAGGTGGCATCAT | TGCCATGTTGCAACTAGCCA | 59.45 | (AG)7 | 8 | 0.23 | 0.84 | 0.82 | No |
| 419 | TGCACAACTCAAACAAACACA | TCTCCTCTTTGCTAACGCCG | 57.35 | (GA)9 | 6 | 0.10 | 0.69 | 0.65 | Yes |
| 421 | CTTCTGCCAGTGAAGTCAGT | GCTGCTCAGTATCTGCTGGA | 57.46 | (TG)9 | 2 | 1.00 | 0.50 | 0.38 | No |
| 422 | TGGAGGTGGAGGTGGTAGAG | GCTCCACCTAGCTAGCTGC | 59.96 | (TTGT)5 | 6 | 0.20 | 0.72 | 0.67 | No |
| 424 | CGTAGTCAAAATGTGGTTGTCCA | ACAGCCTCTGCAAGAAAGGT | 59.44 | (TTTA)5 | 4 | 0.10 | 0.73 | 0.68 | Yes |

**Continue Supplemental Table 3 Polymorphism analysis of 114 novel EST-SSR markers in *Melilotus***

| Primer code | Forward primer (5'-3') | Reverse primer (5'-3') | Tm (°C) | Repeats | *N_A_* | *H_O_* | *H_E_* | *PIC* | Transferability |
| --- | --- | --- | --- | --- | --- | --- | --- | --- | --- |
| 433 | GGTGACCCTGTGACCTGAAC | CCGCCTCAAAACACTTCTGT | 60.25 | (ATG)6 | 7 | 0.05 | 0.84 | 0.82 | Yes |
| 447 | AGCATGAAACTGAGGGGAGA | ACCGAAATGAAAGCCGCAAC | 58.34 | (CA)6 | 5 | 0.76 | 0.79 | 0.76 | Yes |
| 451 | CGCACGAATTGGAAATGGGT | TCCTCGTAAACTGTTGAGGCT | 59.47 | (TCC)5 | 8 | 0.12 | 0.85 | 0.83 | Yes |
| 461 | CCTCCAACACTTCCCTCCAT | GCACTCAGCACCACAATCAC | 59.01 | (TC)7 | 7 | 0.23 | 0.80 | 0.78 | Yes |
| 464 | CCAATCCTTCCATTCGGGCT | GCGGAAGAAAACAAAATGTGTGC | 60.11 | (TAT)6 | 8 | 0.67 | 0.82 | 0.80 | No |
| 472 | ACCCAAAAGTCCCTTCCACC | AGTGCTGATGAGCTTTTCTCCA | 59.81 | (AGT)5 | 4 | 0.13 | 0.66 | 0.60 | Yes |
| 498 | GCCAAATCAGAGTCCCTCAA | ACCAATCAACCTCCAAAAGACA | 57.50 | (ATAA)5 | 6 | 0.00 | 0.76 | 0.73 | No |
| 508 | AGAAATGGATGGGGGAAGAACA | AGCCTAGTTTCCACACACGC | 59.35 | (TG)7 | 4 | 0.08 | 0.64 | 0.57 | Yes |
| 514 | GGTCCTTCGTTGGCTGCTAA | CAGCATCAACAATTCCACCGG | 60.32 | (ATG)5 | 7 | 0.33 | 0.81 | 0.78 | No |
| 528 | TGAAGTCAGGGAAATTGGGATTCT | GGTGACGACGATGACGATGA | 59.96 | (GTA)5 | 4 | 0.40 | 0.72 | 0.67 | No |
| 541 | GCAGAAGCAGAAAGTGGCAA | CGCCTGCCATTCCGGAATAT | 59.33 | (TC)6 | 6 | 0.63 | 0.75 | 0.71 | No |
| 545 | GCCCAGATTGCAAGCTCAAA | ACCAAGGTACCTTCTCTCTCCT | 59.40 | (AG)6 | 6 | 0.92 | 0.75 | 0.71 | Yes |
| 546 | ACTGAGAAAGTGTACTAGGTTTGT | AGGAGAGAAAAATATTGGTGGTGGA | 57.52 | (AG)7 | 5 | 0.40 | 0.75 | 0.71 | No |
| 547 | GGTTGGACAGTGTCTTGGTCA | ATTCATGCAATGGTGGCTCT | 60.13 | (TCT)6 | 6 | 0.10 | 0.59 | 0.56 | Yes |
| 548 | TTTGAATGGCGTGAAGTGCG | TCAAGCCCAGTTGCCCTAAG | 60.04 | (GAC)8 | 5 | 0.07 | 0.75 | 0.71 | Yes |
| Mean |  |  |  |  | 9.58 | 0.28 | 0.81 | 0.79 |  |
